# Genome-wide promoter methylation profiling in a cellular model of melanoma progression reveals markers of malignancy and metastasis that predict melanoma survival

**DOI:** 10.1186/s13148-022-01291-x

**Published:** 2022-05-23

**Authors:** Flávia E. Rius, Debora D. Papaiz, Hatylas F. Z. Azevedo, Ana Luísa P. Ayub, Diogo O. Pessoa, Tiago F. Oliveira, Ana Paula M. Loureiro, Fernando Andrade, André Fujita, Eduardo M. Reis, Christopher E. Mason, Miriam G. Jasiulionis

**Affiliations:** 1 Departamento de Farmacologia, Escola Paulista de Medicina, Universidade Federal de São Paulo, São Paulo, Brazil; 2 Divisão de Urologia, Departamento de Cirurgia, Universidade Federal de São Paulo, São Paulo, Brazil; 3 Departamento de Bioquímica, Instituto de Química, Universidade de São Paulo, São Paulo, Brazil; 4 Departamento de Farmacociências, Universidade Federal de Ciências da Saúde de Porto Alegre, São Paulo, Brazil; 5 Departamento de Análises Clínicas e Toxicológicas, Faculdade de Ciências Farmacêuticas, Universidade de São Paulo, São Paulo, Brazil; 6 Bioinformatics Graduate Program, Instituto de Matemática e Estatística, Universidade de São Paulo, São Paulo, Brazil; 7 Department of Biology, Loyola University Chicago, Chicago, USA; 8 Departamento de Ciências da Computação, Instituto de Matemática e Estatística, Universidade de São Paulo, São Paulo, Brazil; 9 Department of Physiology and Biophysics, Weill Cornell Medicine, New York, USA; 10 Departamento de Farmacologia, Escola Paulista de Medicina, Universidade Federal de São Paulo, São Paulo, 04039-032 Brazil

**Keywords:** Epigenetics, Melanoma, DNA methylation, Biomarkers, Prognosis

## Abstract

The epigenetic changes associated with melanoma progression to advanced and metastatic stages are still poorly understood. To shed light on the CpG methylation dynamics during melanoma development, we analyzed the methylome profiles of a four-stage cell line model of melanoma progression: non-tumorigenic melanocytes (melan-a), premalignant melanocytes (4C), non-metastatic melanoma cells (4C11−), and metastatic melanoma cells (4C11+). We identified 540 hypo- and 37 hypermethylated gene promoters that together characterized a malignancy signature, and 646 hypo- and 520 hypermethylated promoters that distinguished a metastasis signature. Differentially methylated genes from these signatures were correlated with overall survival using TCGA-SKCM methylation data. Moreover, multivariate Cox analyses with LASSO regularization identified panels of 33 and 31 CpGs, respectively, from the malignancy and metastasis signatures that predicted poor survival. We found a concordant relationship between DNA methylation and transcriptional levels for genes from the malignancy (*Pyroxd2* and *Ptgfrn*) and metastasis (*Arnt2*, *Igfbp4* and *Ptprf*) signatures, which were both also correlated with melanoma prognosis. Altogether, this study reveals novel CpGs methylation markers associated with malignancy and metastasis that collectively could improve the survival prediction of melanoma patients.

**Supplementary Information:**

The online version contains supplementary material available at 10.1186/s13148-022-01291-x.

## Introduction

Cutaneous melanoma is a type of skin cancer arising from the malignant transformation of melanocytes. According to the World Health Organization, the worldwide incidence of melanoma surpasses 132,000 cases per year. Despite melanoma having a low incidence compared to other types of skin cancer, its aggressive behavior and potential to develop metastasis results in a high incidence of deaths when detected in later stages [1, 2].

DNA methylation is a well-studied epigenetic mark characterized by the incorporation of a methyl group to cytosines by DNA methyltransferases (DNMTs). In cancer cells, abnormal methylation patterns can lead to genome instability, oncogene expression, and silencing of tumor suppressor genes. While cancer cells are characterized by global DNA hypomethylation, the hypermethylation of gene promoters has an essential role in gene silencing and impaired cell proliferation [6]. In melanoma, epigenetic abnormalities occur along with genetic alterations. Changes in methylation patterns have already been described, including hypermethylation of tumor suppressor genes such as *PTEN* and *RAR-b2*, and hypomethylation of repetitive elements and oncogenes, such as *MAGE* and *GAGE* families of cancer-testis genes [7–9]. Besides the existing DNA methylation data in melanoma, the dynamics of those alterations along tumoral progression is still widely unknown.

Studies using melanoma tumor samples have the downside of including stromal and infiltrating immune cells along with melanoma cells, which can mask the discovery of specific cancer cell biomarkers [12, 13]. Even studies conducted in recent years using single-cell technology have not yet achieved a good resolution and scalability to detect genome alterations that could be achieved only by using bulk methods [14]. To get insights into the dynamic alterations of DNA methylation occurring along with melanoma progression, we have studied epigenetic changes using a four-stage cellular model developed by our group [15, 16]. This model comprises different cell lines representing the main steps of melanoma progression: non-tumorigenic melanocytes (melan-a), premalignant melanocytes (4C), non-metastatic (4C11−), and metastatic (4C11+) melanoma cells. The in vitro characterization of the model has found that the 4C cell line resembles premalignant melanocytes (i.e., non-transformed, mesenchymal-like and undifferentiated cells), whereas 4C11− are non-metastatic, mesenchymal-like and undifferentiated melanoma cells, and 4C11+ as metastatic, highly proliferative and differentiated melanoma cells [17–22, summarized information may be found in the Table S8 in [16]]. A progressive global DNA hypomethylation was observed during melan-a malignant transformation, and both protein level and mRNA expression of DNMTs were previously shown to be altered among cell lines [21]. These results show that epigenetic marks are involved in the malignant transformation of melanocytes in this model [16]. Corroborating our findings, Preston-Alp and colleagues revealed DNA methylation as a key molecular mechanism of melanomagenesis induced by UV radiation (UVR) [23]. The authors showed that UVR directly causes stable changes in the DNA methylome and transcriptome, which affect signaling pathways with role in melanocyte biology. More importantly, these alterations correlate to methylation changes observed in melanoma.

Considering the potential of this melanoma progression model to better understand the malignant transformation of melanocytes up to intermediate and metastatic stages, we analyzed here the genome-wide methylation patterns of CpG-rich areas of the four cell lines using the method of Enhanced Reduced Representation Bisulfite Sequencing (ERRBS) [24]. Then, we evaluated CpG sites in promoter regions to discover epigenetically regulated genes associated with malignancy and metastasis. To identify these alterations, we have taken two approaches: first, we compared the CpG methylation profile of melan-a cells with those of 4C, 4C11− and 4C11+ to identify genes altered during the malignant transformation of melanocytes (malignancy signature). Second, we identified CpG markers and genes that could be specifically related to the aggressive features of metastatic cell line 4C11+ (metastasis signature). Using these signatures, we have conducted a multivariate survival analysis using the TCGA-SKCM cohort and found 140 genes presenting at least 2 CpGs as potential methylation biomarkers for melanoma patients’ prognosis. Furthermore, we discovered CpG methylation panels which provide risk scores with prognostic value for melanoma malignancy and metastasization. In summary, we have identified potential genes regulated by CpG methylation and, more importantly, CpG methylation signatures with potential prognostic value by providing risk scores associated with melanoma malignancy and metastasis.

## Results

### DNA methylation profiling of cell lines corresponding to different stages of melanoma progression

The DNA methylation profiling of the cell lines in the model—melan-a, non-tumorigenic melanocytes [25]; 4C, premalignant melanocytes; 4C11−, non-metastatic melanoma cells; and 4C11+, metastatic melanoma cells [15, 16, 20]—was performed using ERRBS [24], a technique developed to obtain the methylation status of CpGs at base-pair level, with a focus in CpG islands and shores. An average of 1,406,315 cytosines (with a standard deviation of 31,656) in CpG context at minimum 10X coverage was uncovered for each sample. The mapping to the bisulfite converted mouse genome (mm10 assembly) using Bismark [26] had an efficiency of 71% on average. As expected, a bimodal distribution of the methylation status was observed for all cell lines, with peaks in 0 and 100% methylation (Additional file 7: Figure S1).

**Fig. 1:**
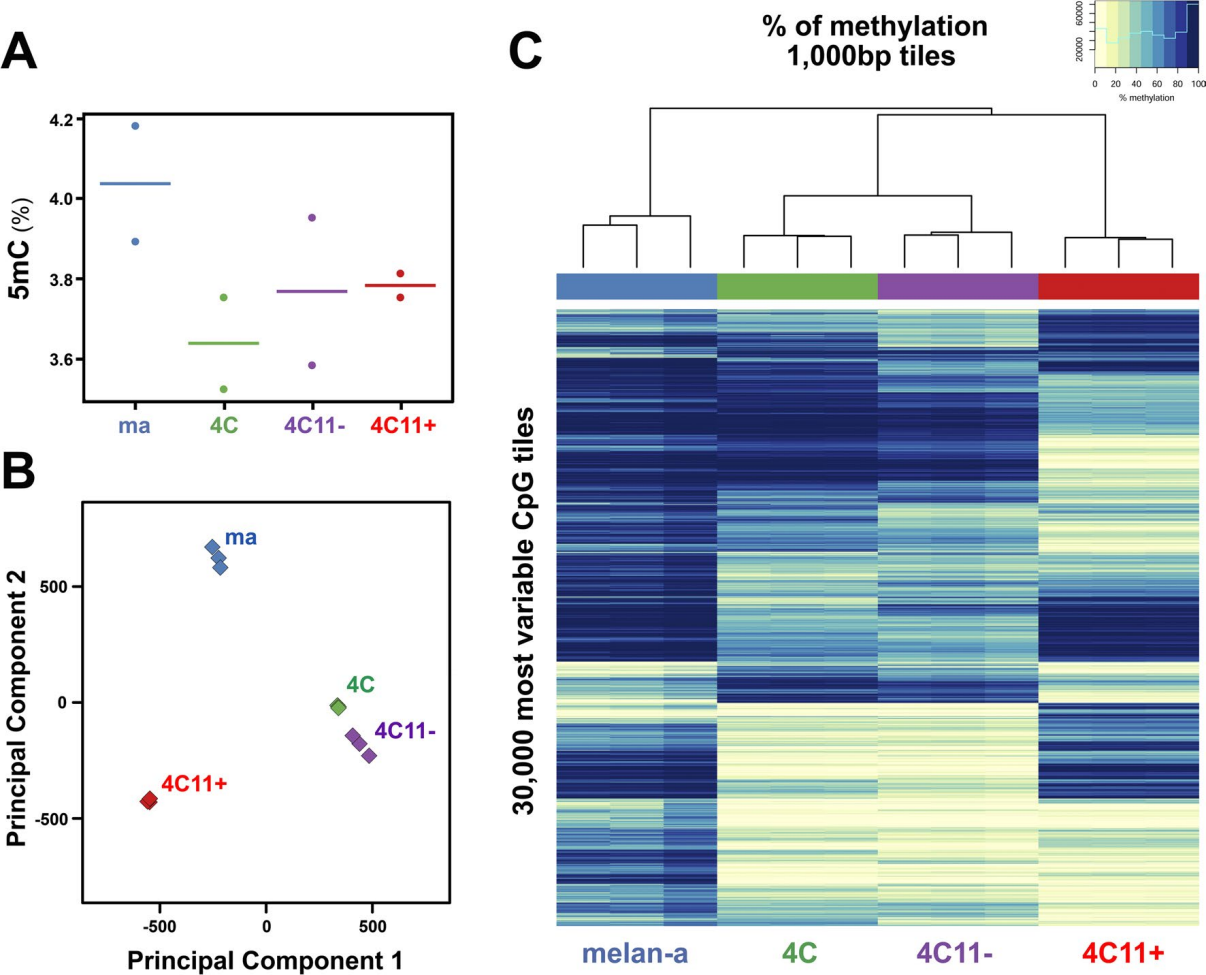
Distinct methylation patterns characterize cell lines representing melanoma progression. **A.** Global DNA methylation content analyzed by HPLC shows the percentage of 5-methylcytosine in the genome for two biological replicates from the cell lines melan-a, 4C, 4C11−, 4C11+. **B.** Principal component analysis of DNA methylation data from three biological samples of the melan-a, 4C, 4C11− and 4C11+ cell lines shows close values of Principal Components 1 and 2 for samples from the same cell line. **C.** A heatmap represented with light yellow for regions with low methylation and dark blue for regions with high methylation shows the top most variant regions of 1,000 bp for each sample. Unsupervised hierarchical clustering in the upper part of the heatmap groups 4C and 4C11− in a common branch closer to 4C11 + than melan-a melanocytes. melan-a: non-tumorigenic melanocyte lineage; 4C: premalignant melanocyte lineage; 4C11−: non-metastatic melanoma cell line; 4C11 + : metastatic melanoma cell line

A methylation average of 47.4% was obtained among CpGs for melan-a melanocytes, whereas 35.8% for 4C, 34.2% for 4C11−, and 36.8% for 4C11+ cell lines. A similar pattern was obtained when analyzing the global content of 5-methylcytosines by HPLC, as shown in Fig. 1A. Methylated cytosines are averaged in 4% of the genome for melan-a lineage and 3.8% for the other cell lines (ANOVA test F = 1.273, p-value = 0.38). The principal component analysis of these data (Fig. 1B) effectively clustered the biological replicates from the same cell lines. Also, it revealed that the DNA methylation status discriminates each cell line from our model, with pre-malignant 4C and malignant non-metastatic 4C11− cell lines displaying similar overall methylation values. This could also be observed by the patterns shown in the heatmap (Fig. 1C), which also include an unsupervised hierarchical clustering that clear stratifies the samples based on their respective cell lines, and also clusters together 4C and 4C11− cells, the two cell lines with similar undifferentiated/mesenchymal phenotype [21].

### The distribution of CpG methylation across the genome in different stages of melanoma progression

**Fig. 2:**
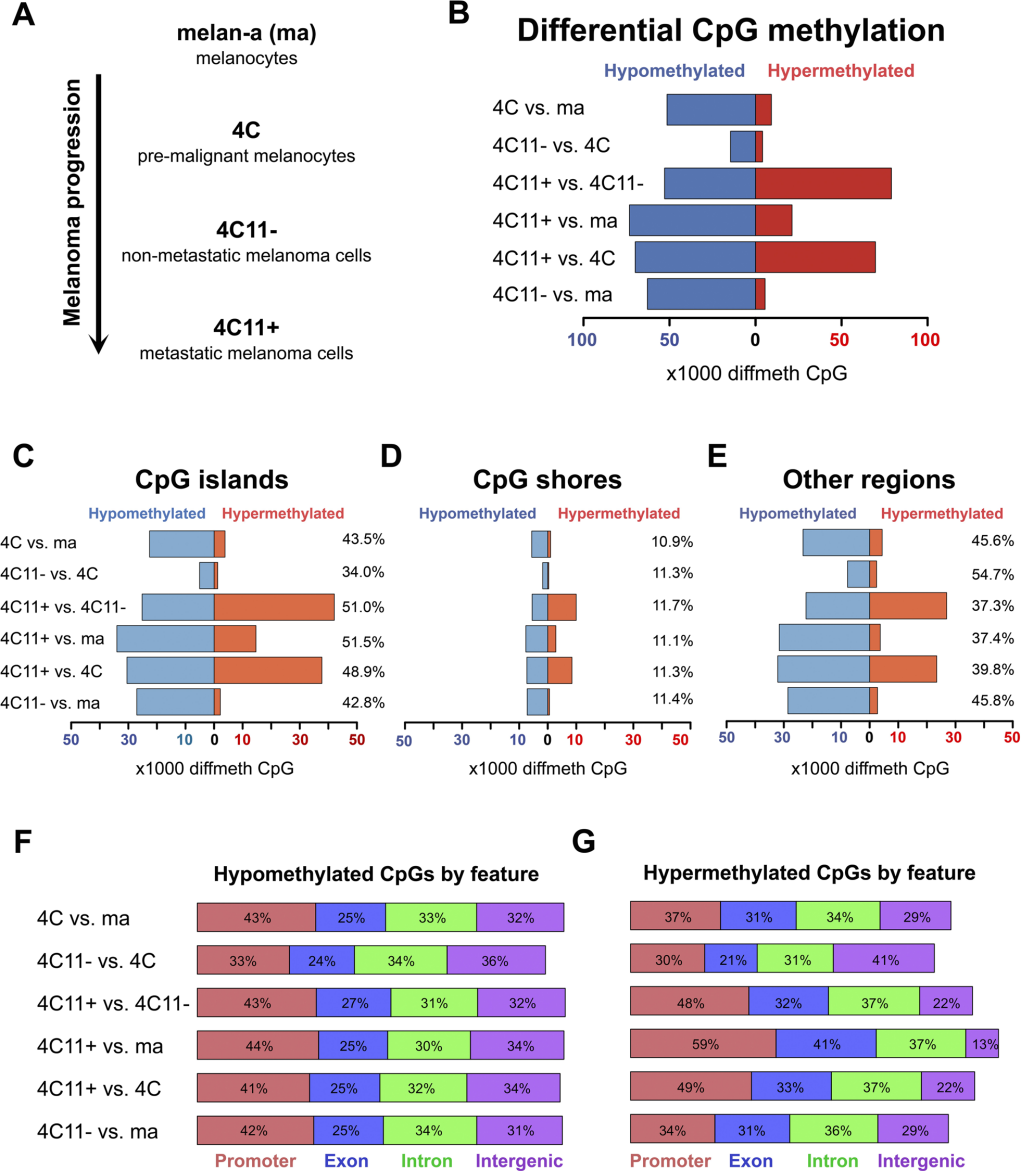
The pattern of differentially methylated CpGs distributed across genomic features and transcription features. **A** Cell lines used in this study that represent different stages of melanoma progression. **B** Number of hypermethylated (red) and hypomethylated (blue) individual cytosines in CpG context per pairwise comparison among all the four cell lines. DMCs according to their location in islands, shores, and other regions are represented in **C**, **D**, and **E**, respectively. Annotation of all hypomethylated (**F**) and hypermethylated (**G**) CpGs by region related to transcription: promoter (salmon), exon (blue), intron (green) and intergenic (purple) for each pairwise comparison

The differential methylation analysis was conducted using the package methylKit [27] in the R environment [28], with multiple hypotheses testing correction by the SLIM method [29]. CpGs with a q-value less than 0.01 and percentage of differential methylation higher than 25% were selected. An overview of the comparative results between the four cell lines from the model can be seen in Fig. 2 and Additional file 8: Figure S2. Additional file 8: Figure S2 contains the heatmaps for pairwise comparisons between the cell lines. A scheme of melanoma model progression can be seen in Fig. 2A, depicting the stepwise transformation of the cells starting from the non-tumorigenic melanocytes melan-a. The comparison between 4C, the premalignant melanocyte lineage and melan-a, the non-tumorigenic parental melanocyte lineage, showed more hypomethylated (51,563) than hypermethylated CpGs (9,190) (Fig. 2B), both equally distributed between CpG islands and other regions, and less among CpG shores (Fig. 2C, D, E, respectively). In parallel, the comparison between the intermediate stages of the model, i.e., between non-metastatic melanoma 4C11− and premalignant 4C cell lines, which display epithelial-to-mesenchymal transition features, had the lowest number of differentially methylated cytosines (DMCs) (18,549) (Fig. 2B). DMCs in other regions of the genome not including CpG islands and shores were more frequent seen in this comparison (Fig. 2C, D, E). Finally, the pairwise comparison between the metastatic 4C11+ and the non-metastatic 4C11− melanoma cell lines revealed the highest number of DMCs (131,202) (Fig. 2B). More than half of the hypermethylated cytosines (41,912) were located in CpG islands (Fig. 2C, D, E).

The comparison between melan-a and 4C11+ cells displayed 22.4% of differentially hypermethylated CpGs (21,092) (Fig. 2B), most of them located in CpG islands (14,592) (Fig. 2C, D, E). The comparison between 4 and 4C11+ cells showed similar results to those obtained in the comparison between 4C11− and 4C11+ (140,261 DMCs). An even distribution between CpGs that gained or lost methylation was observed in this comparison, with the hypermethylated CpGs concentrated in CpG islands (Fig. 2C, D, EE). Last, the pairwise comparison between the 4C11− cell line and melan-a cells revealed a similar pattern to the one found between melan-a and 4C cells. Most differentially methylated CpGs found were hypomethylated in 4C11− cells (Fig. 2B) and only ~ 11% were located in shores (Fig. 2C, D, E).

We also categorized DMCs according to their transcript annotation (promoter, exon, intron or intergenic). The sum of the percentages shown in Figs. 2F (hypomethylated CpGs) and 2G (hypermethylated CpGs) does not total 100% because there is an overlap between transcriptional regions annotated in the genome (for example, between first exons and promoters).

For hypomethylated CpGs, we observed a similar distribution of differential CpGs in the promoter, exon, intron, and intergenic regions across all comparisons, except for the comparison between 4C11− and 4C (Fig. 2F). All comparisons showed a higher frequency of differential CpGs in the promoter regions (41–43%), except for the comparison between 4C11− and 4C, in which they were more located at intergenic regions. For the hypermethylated CpGs, there was a more heterogeneous distribution of CpGs across the transcript annotations, with the most considerable portion of CpGs annotated to promoter regions in all comparisons against the metastasis-prone 4C11 + cell line. In particular, the pairwise comparison between melan-a and 4C11 + cells exhibited the most extensive annotation of differentially methylated CpGs is in promoter (59%) and exon regions (41%). These exon regions probably comprise the first exons of the related transcripts, which also include promoter regions.

### Genes characterized by differential CpG promoter methylation during melanoma progression

We next decided to identify potential genes associated with melanoma progression that could be epigenetically regulated by CpG methylation in promoter regions. The CpGs located in the region from -500 to 500 nucleotides surrounding the TSS (transcription start site) and genes with more than three differentially methylated CpGs were considered in the selection of the relevant genes. The genes with differentially methylated CpGs in their promoter regions are referred hereafter as differentially methylated genes. The complete list of identified genes and CpG sites can be found in Additional file 1: Table S1.

**Fig. 3:**
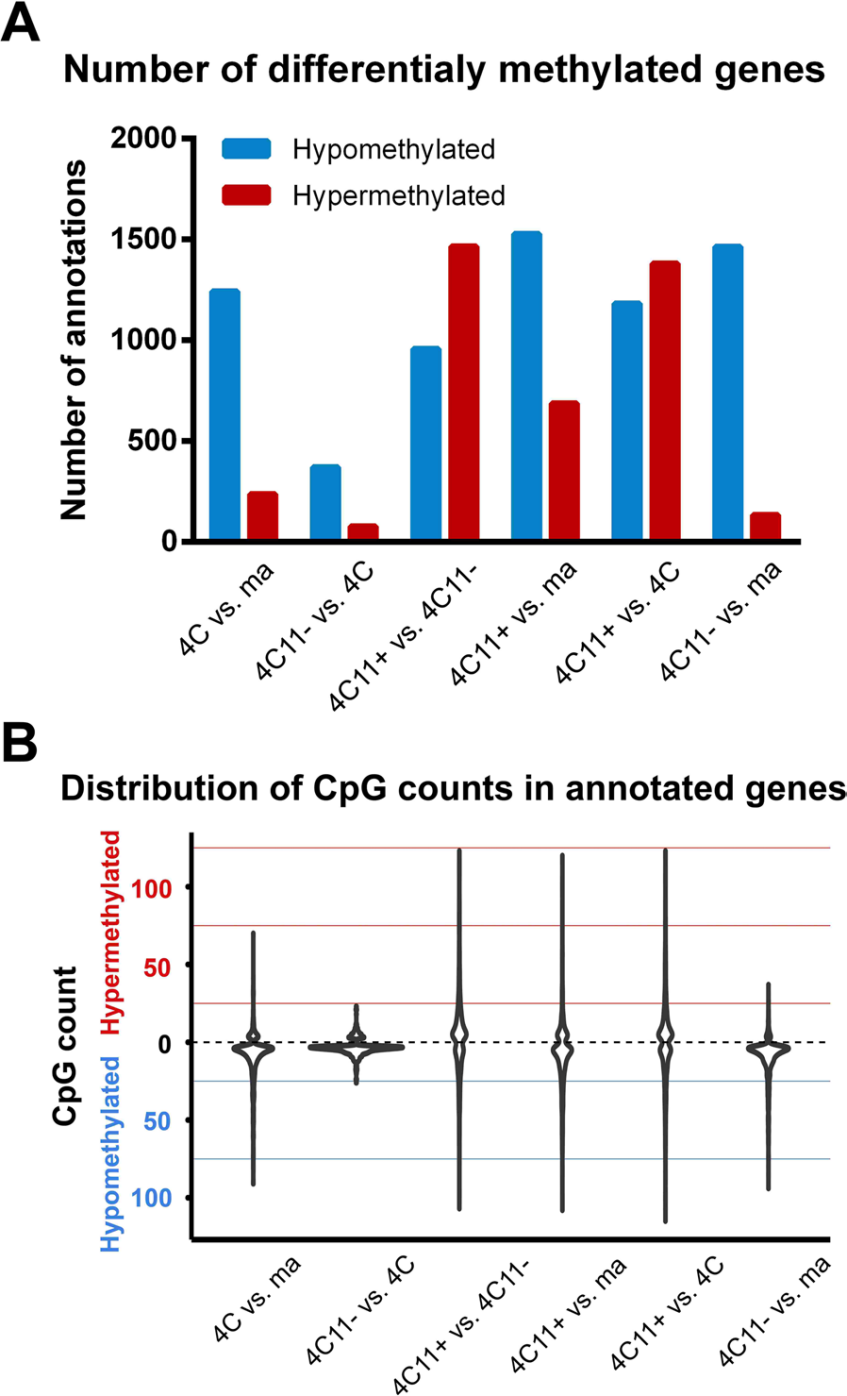
Genes and CpG sites annotated as differentially methylated in each pairwise comparison. **A** Number of hypermethylated and hypomethylated genes per pairwise comparison. Differentially methylated genes were considered as those with differentially methylated CpG sites between 500 bp up and downstream of the TSS, and a minimum of three differentially methylated CpGs. **B** Frequencies of CpG count per gene for each pairwise comparison, with peaks around five cytosines having the greatest number of differentially methylated genes

The number of hypermethylated and hypomethylated genes in each pairwise comparison is indicated in Fig. 3A; these numbers have followed the same pattern observed in Fig. 2A across the same pairwise comparisons. In addition, the distribution of the number of CpGs in each annotated gene at each pairwise comparison can be seen in Fig. 3B. All pairwise comparisons containing the metastatic 4C11 + cell had few genes with more than 100 CpGs hyper- or hypomethylated.

### Gene signatures associated with DNA methylation changes occurring in early and late stages of melanoma progression

**Fig. 4:**
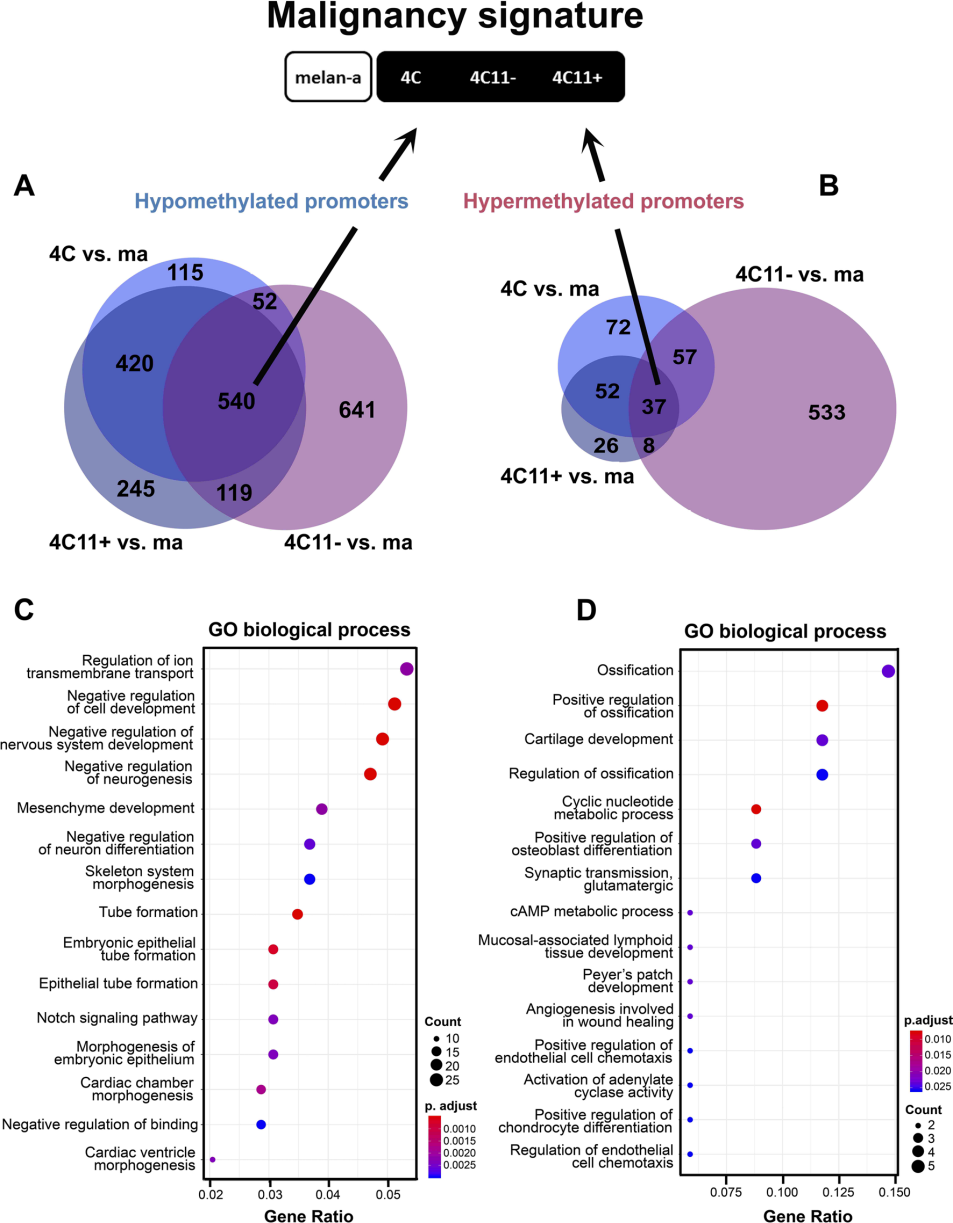
DNA methylation signature associated with melanocyte malignant transformation (malignancy signature). Venn diagram of hypomethylated (**A**) and hypermethylated (**B**) genes in common between pairwise comparisons of 4C, 4C11− and 4C11 + versus melan-a cell line. Enrichment plots showing the pathways enriched in Gene Ontology: Biological Process for hypomethylated (**C**) and hypermethylated (**D**) genes

We sought to identify gene signatures associated with promoter methylation alterations occurring early in malignant transformation (4C pre-malignant cells) and lasting until late stages of melanoma progression (4C11− and 4C11 + melanoma cells). To accomplish that, we conducted an intersection analysis of the differentially methylated genes identified in the pairwise comparisons against melan-a to identify a malignant transformation signature. Five hundred and forty hypomethylated genes and 37 hypermethylated common genes were identified in this analysis (Fig. 4A, B). Functional enrichment analyses were conducted using the software clusterProfiler [30] and the database Gene Ontology (Biological Process), and significantly enriched functions were selected based on FDR corrected p-values. Among the hypomethylated genes, the most significantly enriched functions were related to negative regulation of cell development, nervous system development, and neurogenesis (Fig. 4C), which indicate the role of epigenetic regulation in cell dedifferentiation that occurs along melanoma progression. Moreover, the most significantly enriched functions among the hypermethylated genes were involved in ossification, cartilage development and cyclic nucleotide metabolic process (Fig. 4D).

**Fig. 5:**
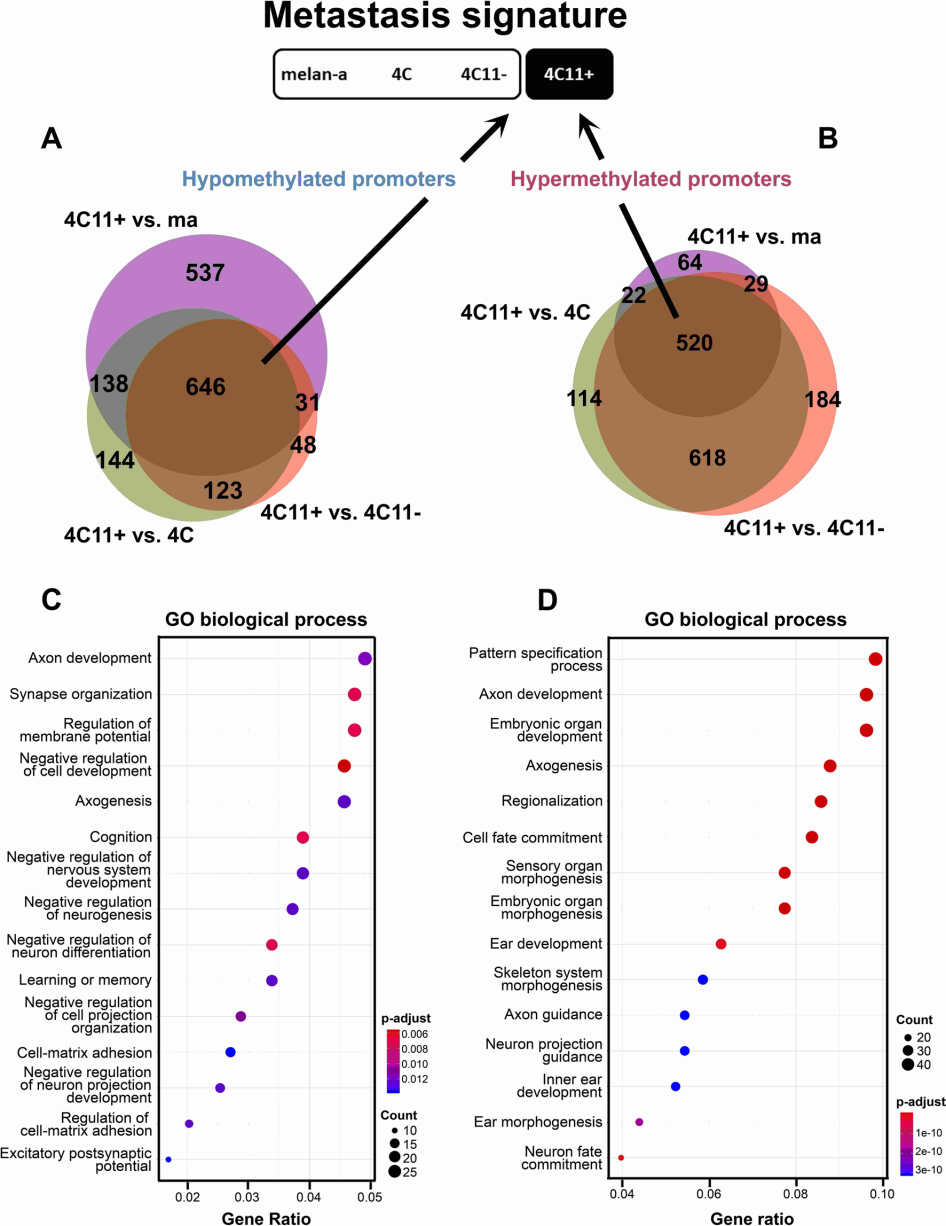
DNA methylation signature associated with metastasis (metastasis signature). Venn diagrams of hypomethylated (**A**) and hypermethylated (**B**) genes only in the metastatic 4C11 + melanoma cell line compared to non-metastatic melan-a, 4C and 4C11− cell lines. Enrichment plots showing the pathways enriched in Gene Ontology: Biological Process for hypomethylated (**C**) and hypermethylated (**D**) genes

In parallel, we decided to investigate hyper- and hypomethylated genes in the metastatic 4C11 + cell line compared to the other three non-metastatic cell lines (melan-a, 4C and 4C11−), in the attempt to find genes potentially related to metastasis acquisition (metastasis signature). Six hundred and forty-six hypomethylated (Fig. 5A) and 520 hypermethylated (Fig. 5B) genes were found in common between the comparisons 4C11− vs. 4C11 + , melan-a vs. 4C11 + and 4C vs. 4C11 + . Independent functional enrichment analyses were also conducted for the hypomethylated (Fig. 5C) and hypermethylated (Fig. 5D) genes. Among the hypermethylated genes, significantly enriched GO terms were associated with nervous system development: axon development, axonogenesis, axon guidance, neuron projection guidance, and neuron fate commitment. Conversely, the hypomethylated genes in the metastasis signature were related to cell–matrix adhesion, as well as to the negative regulation of nervous system development.

### DNA methylation alterations as independent prognostic markers for melanoma survival

**Fig. 6:**
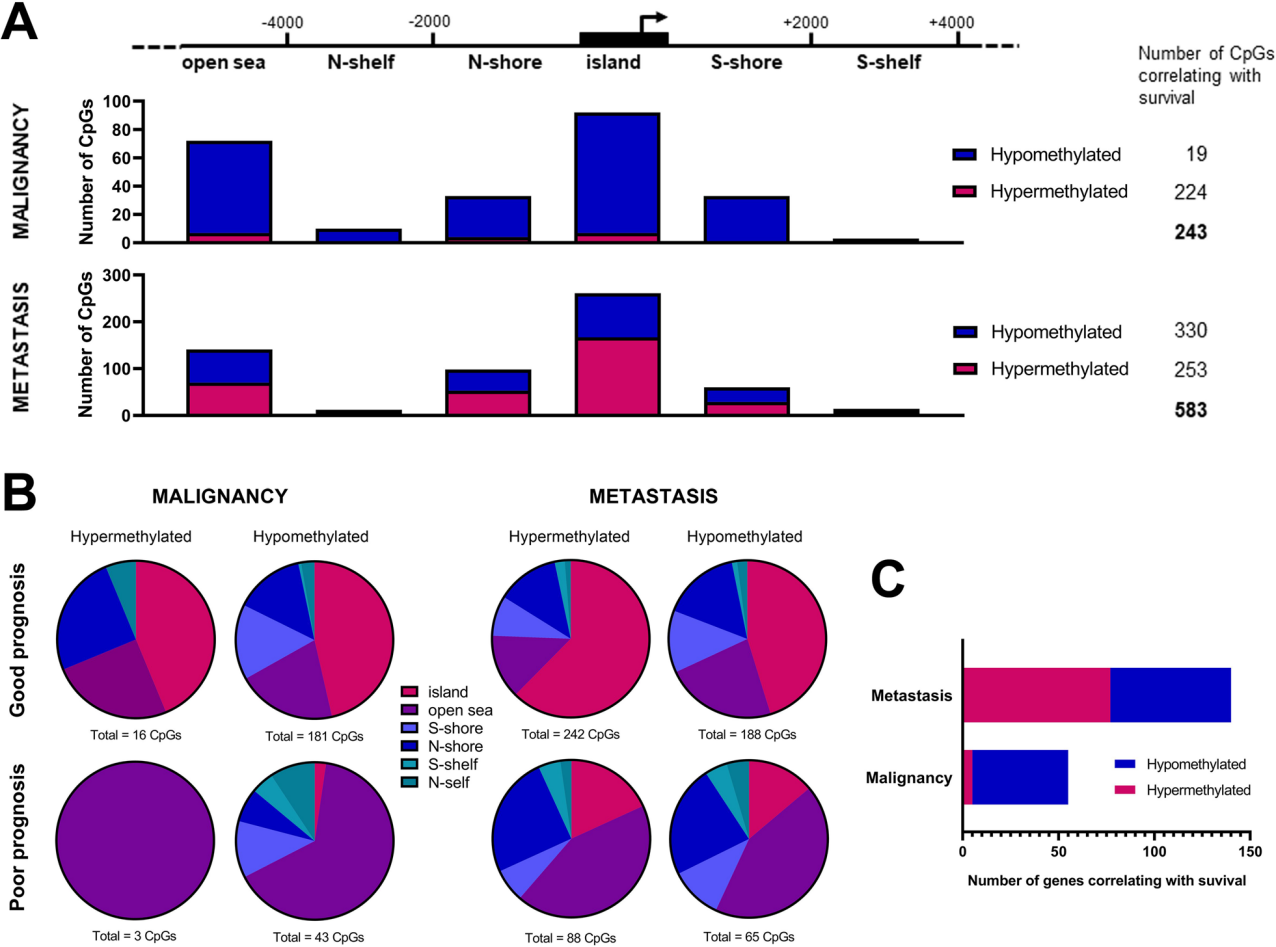
Distribution of hyper- and hypomethylated CpGs correlated with melanoma patients’ outcomes according to their genome location and number of associated genes. **A** The number of hyper- and hypomethylated CpGs correlated with overall survival is shown according to their location in islands, shores, shelves and open sea regions, for malignancy and metastasis signatures. **B** The distribution of hyper- and hypomethylated CpGs along genomic regions is shown according to their correlation with a good or poor prognosis for malignancy and metastasis signatures. **C.** The number of genes from both signatures containing at least two CpGs correlating with melanoma patient outcome is shown

To further investigate the relationships between the DMCs and their potentially regulated genes identified in the murine model with melanoma patient data, we performed overall survival analyses using cutaneous melanoma data from TCGA (TCGA-SKCM). Additional file 6: Table S6 contains a list of all ERRBS CpGs annotated to mouse genes and the ortholog human gene with the respective Infinium 450 k CpG probes. Using the methSurv tool, we obtained Hazard Ratios (HR) and respective p-values for all the TCGA-SKCM's CpGs annotated to the ortholog genes to the hyper- and hypomethylated genes in the malignancy and metastasis signatures. We obtained statistically significant CpGs (adjusted p-values ≤ 0.01) associated with overall survival, resulting in 19 hyper- and 224 hypomethylated CpGs in the malignancy signature, and 330 hyper- and 253 hypomethylated CpGs in the metastasis signature (Fig. 6A, and Additional file 2: Table S2).We next analyzed the statistically significant CpGs according to their location in islands, shores, shelves and open seas. Interestingly, the number of hypermethylated CpGs correlating with patients’ survival in the metastasis signature were concentrated in islands (167 CpGs), followed by open seas (70 CpGs) and N- (53 CpGs) and S-shores. This same pattern was seen among the hypomethylated CpGs with prognostic value from the metastasis signature; which were also found enriched in islands (94 CpGs), followed by open seas (71 CpGs), and N- (45 CpGs) and S-shores (31 CpGs). In the malignancy signature, open seas and islands sites hosted the most hypermethylated CpGs (7 CpGs each). In contrast, more hypomethylated CpGs correlating with survival were found in islands (85 CpGs), open seas (65 CpGs) and N- (29 CpGs) and S-shores (33 CpGs) (Fig. 6A). Nevertheless, the differences between regions in each signature were not significant (Fisher test, malignancy: p-value = 0.944, and metastasis p-value = 0.3571).

We also verified if the CpGs with prognostic value would be enriched in specific genomic locations. Interestingly, in both malignancy and metastasis signatures, the hyper- and hypomethylated CpGs significantly correlated with good prognosis were located mainly in islands, whereas those correlating with poor survival were predominantly in open seas (Fisher test, hypermethylated in malignancy: p-value = 0.1837, hypomethylated in malignancy: p-value = 6.243–12, hypermethylated in metastasis: p-value = 0.0004998, and hypomethylated in metastasis: p-value = 2.333–5) (Fig. 6B).

We also explored the genes whose CpG methylation patterns were related to survival in each signature; a subset of genes containing at least 2 CpGs with significant Cox proportional-hazards models was selected in each signature (Fig. 6C). There were 140 (77 hyper- and 63 hypomethylated) genes identified in the metastasis signature and 55 (5 hyper- and 50 hypomethylated) genes identified in the malignancy signature (Additional file 3: Table S3). Then, only those differentially methylated genes present in similar signatures (malignancy or metastasis) based on gene expression levels (Additional file 5: Tables S5 and Additional file 6: Table S6 from [16]) and with an inverse relationship between expression and DNA methylation patterns were chosen for further evaluation. Finally, the analysis of the prognostic value of these genes based on expression levels was also used to guide gene selection using previous information from the Leeds Melanoma Cohort (Table S8 from [16]; European Genome-Phenome Archive accession number EGAS00001002922), which consists of molecular date from 703 treatment-naïve primary melanoma patients.

Based on the above criteria, *TBC1D9* was selected from the hypermethylated subset, and four genes (*P2RX7*, *PRDM1*, *PTGFRN* and *PYROXD2*) were selected from the hypomethylated group in the malignancy signature. In the metastatic signature, six genes were selected from those hypermethylated (*FAM107B*, *LMX1B*, *MKX*, *MYH10*, *PTPRF* and *ZBTB16*), and eight (*ADSSL1*, *ARNT2*, *CHN2*, *DIXDC1*, *IGFBP4*, *LMX1B*, *PCDHGB2* and *TBX15*) from those hypomethylated..

**Fig. 7:**
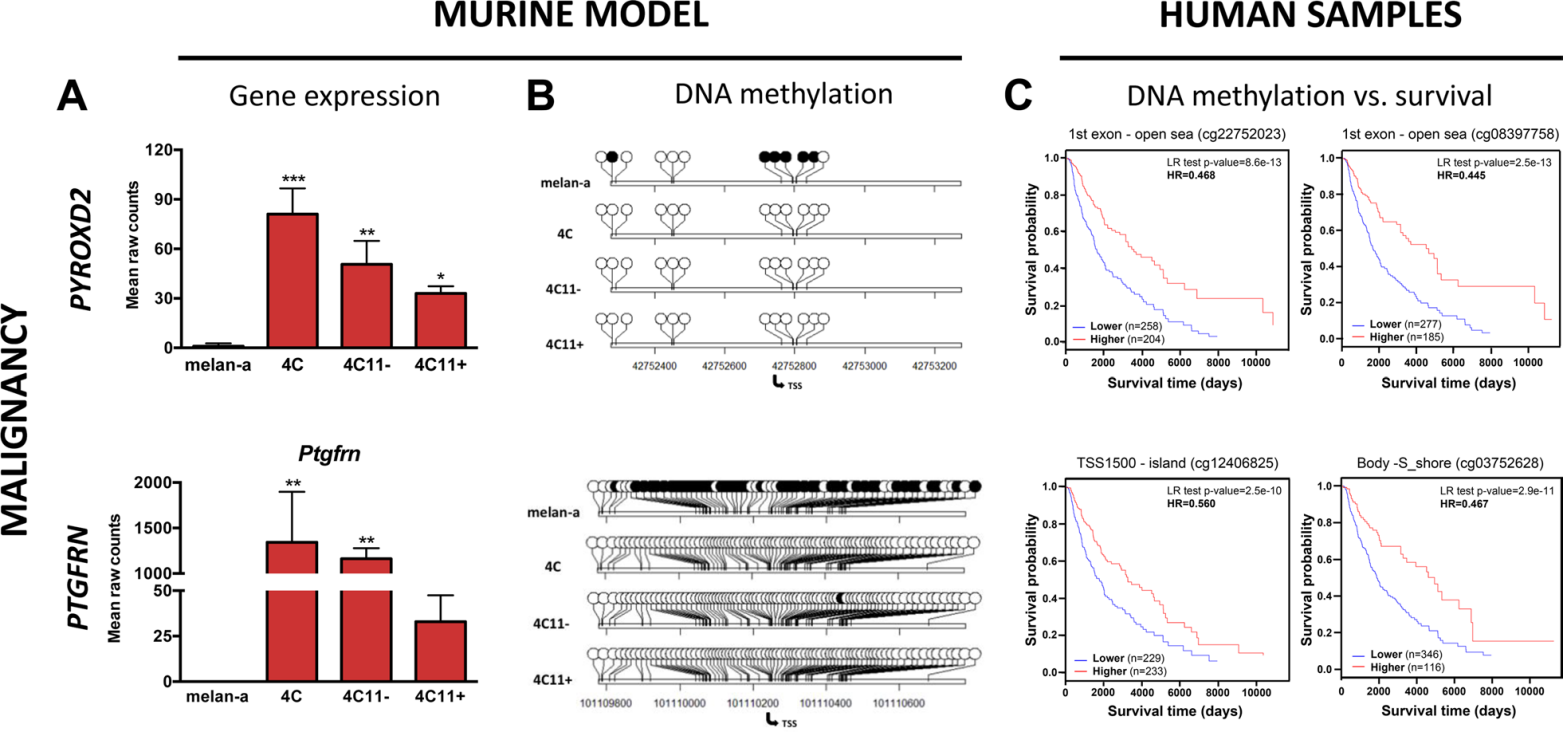
Genes from the malignancy signature presenting CpGs’ methylation status with significant prognostic value. *Pyrodx2* and *Ptgfrn* are shown for gene expression (**A**) and CpG methylation (**B**) in melan-a, 4C, 4C11− and 4C11+ cell lines. **C.** Kaplan–Meier plots show the result of overall survival analysis conducted in patients from the TCGA-SKCM cohort, with samples split by CpG methylation status for CpGs of the respective genes. Gene expression is shown as raw fragment counts, with the bar representing standard error. *p ≤ 0.05, **p ≤ 0.01, ***p ≤ 0.001, ****p ≤ 0.0001 for ANOVA statistical test. CpG methylation sites for the promoter region of the genes are shown as methylated (black circle) for ≥ 70% methylation and white circles for < 70% methylation

No genes from the malignancy signature, both from hypo- or hypermethylation subsets, presented expression levels associated with prognostic value. However, for the metastatic signature, the hypermethylated genes *FAM107B*, *PTPRF* and *ZBTB16* had their low expression correlated with poor overall survival, and the hypomethylated genes *ARNT2*, *IGFBP4* and *CHN2* had their expression levels related to prognostic value. Although no gene from the malignancy signature had its expression correlated with patient survival, we decided to select *PYROXD2* and *PTGFRN* for additional analysis (Fig. 7).

Among the genes in the malignancy signature, we have confirmed that *Pyrodx2* and *Ptgfrn* have increased gene expression in all cell lines compared to melan-a (Fig. 7A), which is in line with the hypomethylation of individual CpGs in their promoter regions in all cell lines compared to melan-a (Fig. 7B). In order to evaluate the effect of DNA methylation on gene expression, the cell lines were treated with the demethylating agent, 5-aza-2’-deoxycytydine (5azaCdR). These genes, highly expressed and hypomethylated in 4C, 4C11− and 4C11− cells compared to melan-a melanocytes, were not derepressed in melan-a cells after 5azaCdR treatment (Additional file 3: Figure S3).

The survival analyses of two CpGs located in the first exons of these genes can be seen in Fig. 7C. Methylation of the *PYRODX2* CpGs identified by the probe IDs cg22752023 and cg08397758 independently classify a group of patients with better survival. The same good prognosis profile is seen for the hypomethylation of *PTGFRN* CpGs cg12406825 and cg03752628, located, respectively, at a distance of 1500 bp from the TSS, in a CpG island, and in the gene body, in a south shore.

**Fig. 8:**
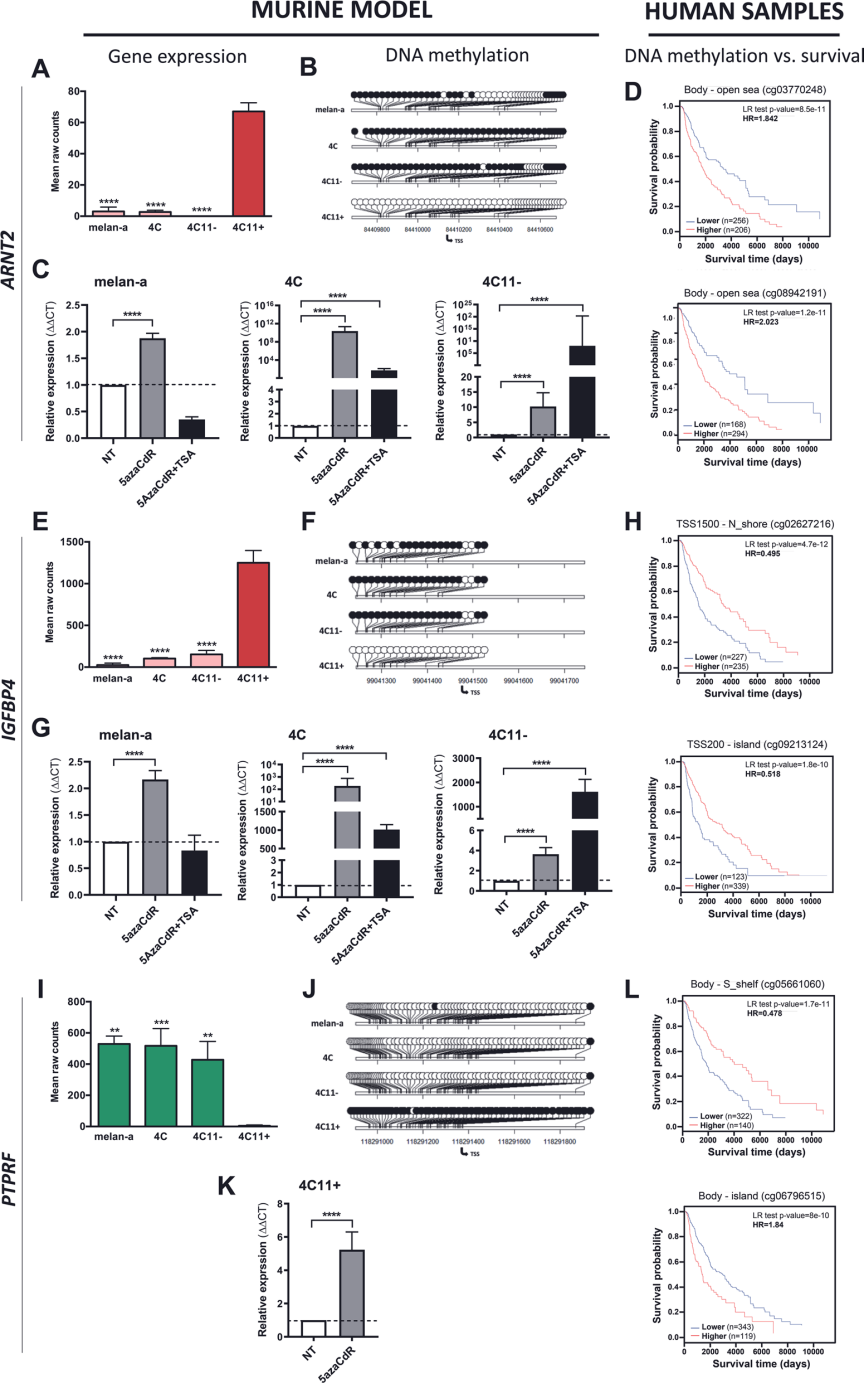
Genes from the metastasis signature with CpGs methylation status correlated with melanoma patient survival. The genes *Arnt2*, *Igfbp4*, and *Ptprf* are shown for gene expression (**A**,** E** and **I**, respectively) and CpG methylation (**B**,** F** and **J**, respectively) in melan-a, 4C, 4C11− and 4C11+ cell lines. **C**,** F** and **K.** Cell lines presenting, respectively, low *Arnt2*, *Igfpb4* and *Ptprf* expression were treated for 48 h only with 5azaCdR or for additional 18 h with Trichostatin A (TSA) and had their expression evaluated by RT-qPCR. **D**, **H** and **L**. Kaplan–Meier plots show the result of overall survival analysis conducted in patients from the TCGA-SKCM cohort, with samples split by CpG methylation status for CpGs of the respective genes. Gene expression is shown as raw fragment counts, with the bar representing standard error

In the metastasis signature, *Arnt2* and *Igfbp4* genes, highly expressed (Fig. 8A) and hypomethylated (Fig. 8B) only in 4C11 + metastatic melanoma cells compared to melan-a, 4C and 4C11− cells, had their expression significantly increased in melan-a, 4C and 4C11− cells after 5azaCdR and 5azaCdR + TSA treatment (Fig. 8C). For both genes, 5azaCdR treatment significantly reverted the gene silencing in melan-a cells, while 5azaCdR + TSA in 4C11 + melanoma cells. In parallel, for *Ptprf* gene, not expressed in metastatic 4C11 + cells compared to melan-a, 4C and 4C11− cells and presenting concordant increased methylation in almost all CpGs located in the promoter region (Fig. 8A, B, respectively), the treatment with 5azaCdR was able to revert gene silencing in 4C11 + cells (Fig. 8C). Survival analyses showed that the hypermethylation of *ARNT2* CpGs in the gene body region correlates with poor survival (Fig. 8D). This is in line with the finding that methylation in the body of this gene is correlated to its higher expression [5; 32–36]. For the *IGFBP4* gene, the hypermethylation of two CpGs, once located in the 1500 bp distance from the TSS, in a north shore, and another located 200 bp of the TSS, inside a CpG island, predicted good patient survival outcomes (Fig. 8D). Interestingly, the methylation status of two CpGs from *PTPRF* exhibited different outcomes for patient survival: cg05661060, located in the gene body, in a shelf region, is hypomethylated in the patient subgroup with poor survival. In contrast, cg06796515 (located on an island) is hypermethylated in patients with poor prognosis.

### Multi-CpG methylation panels of malignancy and metastasis markers discriminate patient prognosis

After analyzing the importance of single CpGs methylation status to patient survival, we decided to investigate the combined prognostic value of a panel of CpG methylation sites and build more accurate Cox proportional-hazard multivariate models for melanoma prognosis based on the malignancy and metastasis CpG signatures. The characteristics of the patients selected for the models are described in Additional file 4: Table S4.

To determine the subset of relevant predictor variables and avoid overfitting in the Cox model, a LASSO regularization step was applied to employ only variables with non-zero coefficients in the final Cox survival model. This final Cox model was built using the survival R package with the selected CpGs as input. The HR was obtained, along with respective coefficients and p-values, corrected using the Benjamini–Hochberg method for multiple testing hypotheses. Furthermore, the significant CpGs (p-adjusted ≤ 0.05) were selected to compose the panels for malignancy and metastasis signatures, totalizing 33 CpGs in the malignancy (Table 1) and 31 CpGs in the metastasis signature (Table 2). The complete set of the CpGs and covariates used in both models can be found in the Additional file 5: Table S5.

**Table 1:** Significant covariables in malignancy signature Cox model

Name	UCSC_RefGene_Name	coef	HR	p.adj
age_at_diagnosis	NA	0.063	1.065	0.000
breslow_thickness_at_diagnosis	NA	0.640	1.896	0.003
cg00033516	*FLJ13197; KLF3*	1.703	5.490	0.000
cg00144186	*FJX1*	− 0.775	0.461	0.043
cg00478851	*PPFIBP2*	1.274	3.575	0.000
cg00489485	*WFDC1*	0.723	2.061	0.020
cg01607369	*PPFIBP2*	− 1.217	0.296	0.001
cg03600687	*BOK*	− 0.910	0.402	0.049
cg04086443	*PHGDH*	0.717	2.048	0.048
cg05358729	*RAB11FIP4*	1.377	3.962	0.013
cg06973225	*SDC3*	− 0.928	0.395	0.049
cg07590102	*CDKL5*	− 1.767	0.171	0.000
cg08034797	*SEPT9*	0.602	1.826	0.043
cg08892078	*KLHDC8A*	− 1.379	0.252	0.001
cg08963581	*ZFHX3*	0.517	1.677	0.043
cg09070371	*HS3ST6*	0.759	2.136	0.043
cg10238675	*CUX2*	0.721	2.056	0.004
cg10581650	*MIR1287; PYROXD2*	0.993	2.699	0.005
cg12368542	*TRIM13; DLEU2*	− 1.342	0.261	0.013
cg13206063	*MICAL1*	− 2.023	0.132	0.000
cg13601997	*LMX1B*	− 1.280	0.278	0.013
cg13786722	*FMN1*	1.166	3.210	0.007
cg13935009	*CDK18*	− 0.936	0.392	0.003
cg16353361	*APLN*	1.087	2.965	0.003
cg17498321	*NOTCH3*	− 0.900	0.406	0.015
cg18189288	*SPATS2L*	0.383	1.467	0.048
cg18329187	*CKB*	1.387	4.001	0.001
cg19802865	*FOXA1*	0.686	1.986	0.007
cg21079003	*RGMA*	1.164	3.203	0.003
cg21642988	*DNAJA4*	− 0.868	0.420	0.007
cg23109191	*GAB2*	0.633	1.883	0.007
cg25244036	*MAPK4*	− 1.475	0.229	0.013
cg25318211	*MICAL1*	1.154	3.171	0.001
cg26604214	*RAP1GAP2*	− 0.840	0.432	0.020
cg27417997	*SHROOM3*	− 0.831	0.436	0.013
gender	NA	− 1.389	0.249	0.021

Table containing CpGs whose methylation was used to compose the risk score of malignancy signature plus the co-variables age, gender, and Breslow depth, all significant from the Cox model of malignancy signature

**Table 2:** Significant covariables in metastasis signature Cox model

Name	UCSC_RefGene_Name	coef	HR	p.adj
age_at_diagnosis	NA	0.130	1.138	0.000
cg00263760	*VAX1*	1.019	2.771	0.008
cg00478851	*PPFIBP2*	0.921	2.511	0.040
cg00562312	*ADAMTS2*	0.817	2.264	0.040
cg00807464	*CUX2*	1.151	3.160	0.005
cg01607369	*PPFIBP2*	1.079	2.943	0.010
cg01965173	*RIPPLY2*	− 2.296	0.101	0.001
cg03818920	*RIPPLY2*	1.587	4.891	0.031
cg03847279	*FGFRL1*	1.547	4.697	0.000
cg05632623	*NPTX1*	− 2.470	0.085	0.004
cg06103654	*DPYSL5*	1.111	3.036	0.004
cg06385227	*EN2*	− 1.416	0.243	0.000
cg09619271	*IGFBP3*	− 0.652	0.521	0.031
cg11317199	*TRIM14*	− 0.637	0.529	0.031
cg12094808	*FRMD5*	− 0.760	0.468	0.031
cg12156672	*CDK5R2*	− 0.922	0.398	0.031
cg12387713	*MSX2*	0.877	2.404	0.007
cg13058819	*SLC35F3*	− 1.230	0.292	0.006
cg14002622	*CDK5R2*	0.833	2.301	0.006
cg15004938	*SYN3; TIMP3*	0.928	2.529	0.031
cg16130019	*JAZF1*	1.029	2.798	0.011
cg16449084	*SSH3; ANKRD13D*	− 0.865	0.421	0.050
cg16603596	*RIMKLB*	− 1.142	0.319	0.028
cg18353826	*OLFM2*	1.069	2.912	0.004
cg19856252	*KY*	1.151	3.161	0.007
cg22897522	*MYH10*	− 1.067	0.344	0.006
cg23123250	*SPNS2*	0.790	2.202	0.031
cg23372684	*COL23A1*	− 0.975	0.377	0.030
cg23894948	*SLC7A4*	1.625	5.080	0.000
cg24647724	*DNAH9*	− 1.634	0.195	0.004
cg25927375	*KIRREL3*	0.916	2.500	0.005
cg26057780	*BDNF*	1.528	4.611	0.006

Table containing CpGs whose methylation was used to compose the risk score of metastasis signature plus the co-variable age, significant from the Cox model of metastasis signature

Interestingly, there were two CpGs in common among the signatures: cg00478851 and cg01607369. The first CpG exhibited the same direction of methylation regulation, while the second showed opposite directions between the two signatures. cg00478851 is present within a distance of 1500 base pairs from the TSS of *PPFIBP2*, in a north shore of the closest CpG island; it has HR values of 3.6 and 2.5, respectively, in the malignancy and metastasis signatures, showing its importance for both tumor initiation and metastasization. On the other hand, cg01607369, which is located in the gene body of *PPFIBP2*, and an open sea region, had an HR of 0.3 in the malignancy but a HR equal of 2.9 in the metastasis signature; it may be concluded that its methylation status is associated with better prognosis during tumor initiation but with worse prognosis at the metastatic state.

**Fig. 9:**
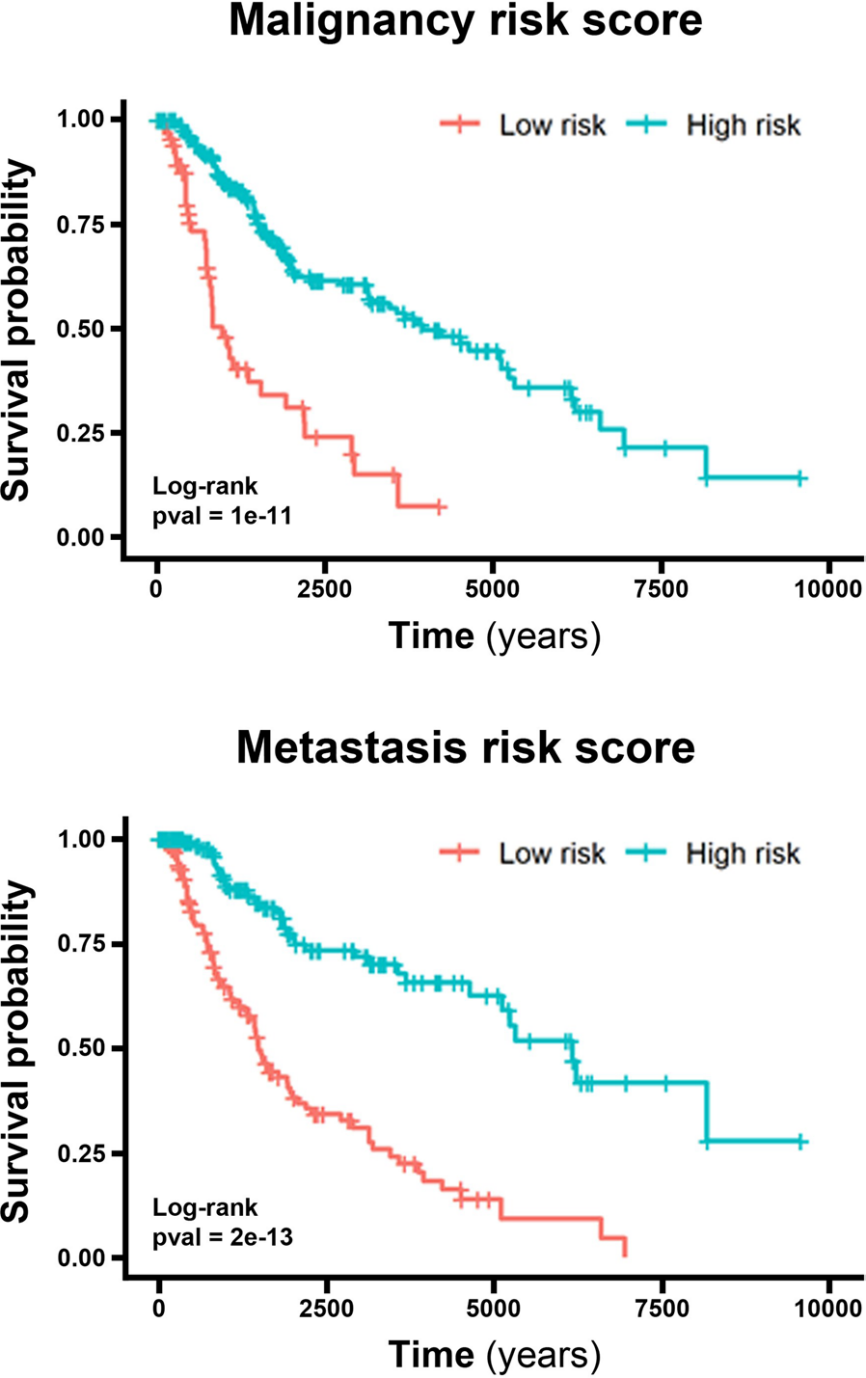
Risk scores for melanoma survival calculated from CpG methylation malignancy and metastasis-related panels accurately separate samples into low and high-risk groups. Kaplan–Meier plots show survival of high and low risk patients according to a (**A**) Malignancy Risk Score (HR = 3.29 [2.48–4.37]), and (**B**) Metastasis Risk Score (HR = 1.84 [1.56–2.16]), both obtained from the respective signatures of CpG methylation status, containing, respectively, 33 and 31 CpGs

The regression coefficients from the CpGs in the Cox multi-CpGs models were used as weights to their respective methylation β-values to calculate a risk score per patient using either the malignancy or metastasis signatures. The Kaplan–Meier survival curves from patients stratified on by high- and low-risk score groups based on the median value are shown in Fig. 9 (log-rank malignancy p-value = 1e-11, metastasis p-value = 2e-13), demonstrating the accuracy of both CpG panels in correctly predicting patient prognosis.

## Discussion

In this work, we analyzed the dynamics of DNA methylation patterns in a murine cellular model of melanoma progression. In addition to genetic and chromosomal alterations occurring throughout melanoma progression, it is well accepted that epigenetic alterations play a role in tumorigenesis by either promoter hypermethylation of tumor suppressor genes or global hypomethylation. These epigenetic changes collectively induce an unstable genomic state, prone to genomic mutations and expression of transposable elements [36–38].

Despite different studies having described DNA methylation alterations in melanomas, few of them have evaluated these alterations in samples or cells with similar genetic backgrounds corresponding to different stages of melanoma progression. To fill this gap, we have performed a differential methylation analysis using pairwise comparisons between cell lines representing distinct stages of a linear model of melanoma progression. This analysis has provided vital information about the CpG methylation dynamics occurring in each step of the melanoma progression.

All cell lines (premalignant 4C, non-metastatic 4C11−, and metastatic 4C11 +) derived from the melan-a melanocyte lineage showed progressively more hypomethylated CpGs concentrated in gene promoters and equally distributed in either CpG islands or regions not including islands or shores (called “open sea”). This observation is in line with the findings from Ecsedi and colleagues, in which decreased promoter methylation was correlated with increased Breslow thickness in melanomas [39]. Opposingly, the hypermethylation of upstream regulatory regions of genes and the hypomethylation of repetitive genome elements have been described in melanoma cell lines corresponding to stages III and IV compared to normal melanocytes [40]. Another study classified melanomas in three groups, presenting high, intermediate, and low methylation in promoter regions. It showed that most of them were categorized in the high methylation group [41]. These contradictory results could be related to cell lines and/or tumor samples corresponding to different melanoma stages. In addition, melanoma tissue specimens, a diversity of patient treatment procedures could contribute to the heterogeneous molecular alterations observed in different studies. Moreover, adjacent tissue and lymphocyte infiltrates can influence the results of studies using tissue biopsies [11, 12].

The comparison between the metastatic melanoma cell line 4C11 + with premalignant melanocytes 4C and non-metastatic melanoma cells 4C11− showed hypermethylated CpGs concentrated mainly in islands and promoter regions. This is consistent with the findings of Wouters et al., in which higher methylation levels were also seen in CpG islands and promoters of metastatic melanomas compared to nevi and primary melanomas [42]. In our model, 1,351 hypermethylated and 848 hypomethylated genes in the comparison between metastatic 4C11 + and non-metastatic 4C11− melanoma cells, which could be related to the acquisition of metastatic capacity.

Early in melanoma progression (4C vs. melan-a), there are many hypomethylated genes, most with a low CpG count (Fig. 3B). In the next transition between premalignant (4C) and non-metastatic melanoma cell line (4C11−), the number of genes presenting alteration in promoter methylation is small. Differences of methylation per gene do not reach a high number of CpGs (Fig. 3). In the metastatic cell line (4C11 +), which represents the latest stage of progression in our model, both hypo- and hypermethylated genes containing high number of DMCs are present compared to its non-metastatic counterpart 4C11−, and also with premalignant 4C and melan-a melanocytes.

In the metastatic stage of our model, there were more genes with DMCs in their promoters. In a previous study, our group showed a higher protein and mRNA expression of the de novo DNA methyltransferase DNMT3a in 4C11 + compared to all the other cell lines. In contrast, the maintenance DNA methyltransferase DNMT1 is highly expressed in all cell lines derived from melan-a melanocytes [21]. Zhang and collaborators have shown that the ectopic expression of DNMT3a in embryonic stem cells leads to the hypermethylation of 5-methylcytosines in the DNA [43]. Therefore, it is possible that the hypermethylation state and increased number of methylated CpGs per gene observed in 4C11 + cells might be induced by the increased DNMT3a expression. Nevertheless, more studies would be necessary to confirm this hypothesis.

Hypermethylated genes in the metastasis signature were enriched with functions related to nervous system development. Both peripheral neurons and melanocytes are cells that originated from the neural crest; therefore, it is reasonable to conceive developmental genes would be regulated by epigenetic reprogramming during melanoma progression. Numerous developmental genes are upregulated in 4C and 4C11− cells in our model, such as *Twist1*, *Snai1*, *Gata4*, and *Nanog* [16]. Moreover, these cells exhibit lower expression of E-cadherin and gain of N-cadherin expression, leading to the conclusion that they undergo cell reprogramming and exhibit a mesenchymal and less differentiated phenotype.

The contribution of each discovered DMCs from the malignancy and metastasis signatures was assessed in the context of melanoma survival, using TCGA-SKCM CpG methylation data [44]. There were more CpGs whose methylation levels were related to good prognosis, both in the malignancy and metastasis signatures. They were predominantly hypomethylated in malignancy, similarly to the findings of de Unamuno Bustos et al., where the unmethylation of promoter CpG islands of multiple genes was associated with good prognosis in a cohort containing predominantly primary melanomas (140 of 170 samples) [45]. In the metastasis signature, the CpGs with good prognostic value were mainly hypermethylated, in line with the findings of Tanemura and colleagues, in which *MINT31* gene CpG island methylation conferred good prognosis in stage III melanoma [46].

We have found a concordant relationship between lower promoter methylation and higher gene expression levels for *Pyroxd2* and *Ptgfrn* from the malignancy signature, which was also associated with the poorest survival in melanoma patients (Fig. 7). This is the first time that the promoter methylation levels of *Pyrodx2* and *Ptgfrn* have been described in melanoma, which could be further explored as novel individual biomarkers for this disease. PYROXD2 is differentially expressed in basal cell carcinoma [47] and is localized in the mitochondrial inner membrane, where it is associated with increased cell proliferation and ATP production [48], highlighting its role in melanoma survival and metabolism.

Notably, *PTGFRN* is overexpressed in glioblastoma multiforme and is associated with poor survival [49]. However, its methylation status has not yet been investigated. Glioblastoma and melanoma are closely related types of cancer, both aggressive and with the same developmental origin from neural crest cells.

We have also investigated the relationship between DNA methylation and gene expression levels for three genes from the metastasis signature (*Arnt2*, *Igfbp4* and *Ptprf*). *Arnt2* expression was increased in the metastasis-prone 4C11 + cells and diminished DNA methylation compared to the other cell lines (melan-a, 4C and 4C11−) (Fig. 8). This gene belongs to the bHLH/PAS transcription factor family, and its promoter has already been demethylated in P19 embryonic carcinoma cells [50]. Besides, its higher expression was associated with breast cancer metastasis [51] and a tumorigenic molecular signature in glioblastoma [52]. Curiously, we have found that methylation alterations in two CpGs at this gene body correlated with poor survival. Indeed, there is consistent evidence about the positive correlation between methylation at a gene body and increased gene expression [31–36].

IGFBP4, a member of the insulin-like growth factor binding protein that regulates angiogenesis [53], exhibited higher gene expression in the 4C11 + cell line and concordantly lower promoter methylation (Fig. 8). Furthermore, in TCGA-SKCM, the analysis of the methylation levels of CpGs close to the TSS was able to identify a subgroup of patients with low methylation and worst survival outcome. There is vast published literature about *Igfbp4* and different types of cancer. Particularly in melanoma, one study compared primary and metastatic tumors and found increased protein levels of IGFBP4 in primary versus metastatic tumors [54]. Elevated transcriptional levels of this gene have also been found in glioblastoma biopsies [55] and lung cancer tissue [56].

Finally, the gene *Ptprf* was found to have a lower expression level in 4C11 + cells and concomitant hypermethylation of its promoter compared to the other cell lines (Fig. 8). Curiously, one study reported the opposite finding, showing that PTPRF was over-expressed in metastatic and primary melanoma cells in vitro and in situ compared to melanocytes [57]. In contrast, its expression was found to be downregulated in non-small cell lung cancer cell lines [58]. The survival analysis of DNA methylation within *PTPRF* revealed a remarkable high HR (1.84) for a CpG located in the gene body in a region classified as a CpG island: patients with lower methylation levels exhibited better survival. This particular CpG methylation shall be better evaluated in the clinic as a potential biomarker of melanoma aggressiveness.

To obtain a more accurate prediction of melanoma survival based on CpG methylation levels, we decided to build panels of CpGs using multivariate Cox regression after LASSO regularization. The final models were composed of a malignancy signature panel with 33 CpGs, and another panel from the metastasis signature with 31 CpGs (Tables 1 and 2). These panels were then applied to calculate survival risk scores and more accurately classify patients into low and high-risk groups (Fig. 9). Wouters and colleagues also identified two signatures associated with different 4-year survival outcomes for melanoma using more than 734 DNA methylation markers [42]. However, survival multivariate analyses were not evaluated for these probes. This multivariate approach has already been conducted for clear cell renal carcinoma [59], leukemia [60], esophageal squamous cell carcinoma [61], colorectal cancer [62], lung adenocarcinoma [63], among others. Future validation of the identified CpGs methylation panels in a new cohort will be deemed necessary for their use in the clinic.

## Conclusions

Our findings demonstrate the usefulness of the melanoma progression cell model to unravel epigenetic changes occurring during melanoma progression. This model can further contribute to discovering molecules present in one specific disease stage, which could be clinically employed to tailor treatment for patients. Furthermore, the CpGs methylation differences identified in this study were practical to identify relevant genes that participate in the control of phenotypic switching along with melanoma progression.

The limitations of our study are related to the fact that ERRBS is a technique that evaluates CpG sites located mostly in CpG islands; therefore, even with our findings of interesting methylation differences in different regions of the genome, the exploration of differential methylation in non-island or shore regions was limited. Furthermore, we focused on the differential methylation analysis for the CpGs located in the promoter region of the genes. Consequently, further studies comparing CpG methylation differences in non-promoter regions could unveil novel results.

The DNA methylation profiling of the melanoma progression steps in our model revealed markers associated with malignancy and metastasis, which were also predictive of human melanoma survival. These markers include DNA methylation changes in *Pyrodx2* and *Ptgfrn*, which are hypermethylated in melan-a non-tumorigenic cells, *Arnt2* and *Igfbp4*, that are hypomethylated in malignant metastatic 4C11 + cells, and *Ptprf*, which is hypermethylated in 4C11 + cells. Furthermore, we established panels of CpGs using multivariate prognostic models that could accurately predict survival outcomes in human patients, and deserve to be evaluated in future studies in search of reliable biomarkers for the clinical assessment of melanoma prognosis.

## Material and methods

### Cell culture

The cell lines 4C, 4C11− and 4C11 + were cultured in RPMI 1640 medium (Gibco) pH 6.9 with addition of 1.0 × 10^5^ U/L antibiotic Pen-Strep (Gibco) and supplement of 5% fetal bovine serum (FBS, Gibco). The cultures were maintained in humidified incubators containing 5% of CO_2_ at 37 °C. Melan-a cells were cultured under the same conditions, but with addition of 200 nM phorbol 12-myristate 13-acetate (PMA, Amresco), an activator of PKC needed for the growth of melanocytes in culture [24]. Three biological replicates of each cell line, obtained from distinct passages, were obtained in the conditions described above.

### Enhanced reduced representation bisulfite sequencing (ERRBS)

To obtain pure samples, DNA was extracted from cultured cells using the Gentra Puregene Cell Kit (Qiagen), according to the fabricant recommendations, including the addition of RNase A solution (contained in the kit). Approximately 200 µL of a minimum concentration of 80 ng/µL were forwarded to the Epigenomics Core Lab of Weill Cornell Medical College, with sufficient quality of double-stranded DNA determined by fluorimetry using QuantiFluor® dsDNA System (Promega), following the Epigenomics Core recommendations. After passing the quality controls, all the samples had their libraries constructed as described by Garrett-Bakelman and colleagues [24]. The samples were multiplexed and distributed in three lanes for sequencing in Illumina HiSeq 2500.

The output files in format.bcl (base call) were demultiplexed and converted to fastq files using the software bcl2fastq. Adaptors and low-quality reads were removed, and the reads were aligned to the mm10 mouse bisulfite converted genome using Bismark [26], to generate CpG calls. These steps were conducted by the Epigenomics Core team.

### Differential methylation analyses

Differential methylation analyses were conducted for each of the pairwise comparisons in the R environment [28], using the package methylKit [27]. From the alignment output file, containing minimum of 10 count coverage, counts above 99.9 percentile were filtered out, to avoid PCR bias. The data were corrected for overdispersion for a more stringent statistical analysis. P-values were corrected to q-values using the package default method: SLIM [29].

The parameters for selection of differentially methylated CpGs (DMCs) were: q-value ≤ 0.01 [29] and minimum of 25% of difference in methylation between compared lineages. Annotation of functional features (promoter, exon, intron, intergenic regions, TSSs), and of CpG classification (CpG islands, shores, and other regions), was conducted using the package Genomation [64]. Reference.bed files were downloaded from the table browser UCSC [65], containing only RefSeq curated genes for functional features, and default CpG islands classification, both based on mm10 genome assembly.

Promoter regions for annotation of genes were defined as within the range from -500 to + 500 base pairs of distance from TSS, and minimum of three DMCs were considered for accounting the gene as having its promoter differentially methylated.

From the annotated genes with differentially methylated promoters, a selection was made based on the cell lines’ characteristics. Hypomethylated and hypermethylated genes present in the intersection among the comparisons between each of the cell lines 4C, 4C11− and 4C11 + with melan-a were gathered as being related to melanocyte malignant transformation, whereas genes present in the intersection among the comparisons between all non-metastatic cell lines (melan-a, 4C and 4C11−) with the aggressive lineage 4C11 + were accounted as related to melanoma metastasis/aggressiveness in the model. Venn Diagrams were constructed using the BioVenn tool [66].

### 5-Methylcytosine content

Nuclear DNA (6 mg), extracted as described in the ERRBS method, was transferred to 54.5 µL of Milli-Q water containing 2.5 µL of 200 mM Tris/MgCl_2_ buffer (pH 7.4) and 1 µL (2.5 units) of deoxyribonuclease I (Qiagen). The samples were incubated at 37 °C for 1 h. Then, 1 µL (0.001 units) of phosphodiesterase I and 1 µL (2 units) of alkaline phosphatase were added, followed by further incubation at 37 °C for 1 h. Aliquots of 50 µL of residual volume were analyzed by a HPLC/PDA system (Shimadzu), as follows: a 250 mm × 4.6 mm i.d., 5 µm, Luna C18(2) column (Phenomenex) was eluted with a gradient of 0.1% formic acid in water (Solution A) and CH_3_OH:H_2_O (1:1, v/v; added of 0.1% formic acid; Solution B; from 0 to 25 min, 0 to 36% B; from 25 to 27 min, 36 to 0% B; from 27 to 37 min, 0% B) at a 1 mL/min flow rate and 30 °C. The PDA detector was set at 286 nm. Calibration curves were constructed at intervals of 0.5 to 8 nmol for 2′-deoxycytidine (dC) and 0.01 to 0.8 nmol for 5-mC. The percentage of global DNA methylation was calculated using the following equation:

$${5mC}_{\left(\%\right)}=\frac{{5mC}_{\left(nmol\right)}x100}{{5mC}_{\left(nmol\right)}+{dC}_{\left(nmol\right)}}$$

### Pathway enrichment

The gene enrichment analysis in the database Gene Ontology: Biological Process of the overlapped genes involved in malignant transformation and melanoma aggressiveness was conducted using the R package clusterProfiler [30]. The enrichment analysis is based on hypergeometric distribution, and it calculates q-value for FDR control.

### Survival analyses per CpG

The Cox proportional-hazard models for overall survival were built using TCGA-SKCM data for all the CpGs annotated to orthologs of the differentially methylated genes from the malignant transformation and melanoma metastasis signatures, using the methSurv tools developed by Modhukur et al. [44]. In methSurv, the analysis is made by individual CpG sites, and patient subgroups are separated based on methylation status into high and low methylation according to distinct parameters, such as median, mean, first quartile (q25) and fourth quartile (q75). Each model contains the covariables: age, sex, and a single CpG methylation. Kaplan–Meier plots of selected CpGs were built using the website provided by methSurv authors, which is https://biit.cs.ut.ee/methsurv/. All plots were generated splitting the samples by the best method, with log-rank p-value accounted, and adjusted for the covariates age and sex. All models with adjusted p-values ≤ 0.01 are represented in the Additional file 4: Table S4.

### Survival analyses with multiple CpGs

The CpGs whose methylation patterns were significantly associated with survival in the Cox models (single CpG per model) for each signature were selected and combined in a new multivariate model with multiple CpGs. LASSO regularization was performed in the Cox proportional-hazards models for selecting variables and avoiding overfitting. The glmnet R package was used, with minimum λ obtained after 20 simulations. After regularization, a multivariate Cox regression was performed, using the survival R package and CpGs methylation, gender, age, and Breslow depth as covariates. The coefficients from the significant CpGs in the Cox models were used in to obtain a risk score for each patient: ${\alpha }_{j}=\sum {\varphi }_{i}\times {\beta }_{ij}$, where φ is the coefficient in the Cox model for the ith CpG, β is the methylation β value for the ith CpG in the j^th^ patient, and α is the risk score for the jth patient. The samples were split based on the mean risk score value in high and low risk subgroups, and Kaplan–Meier plots were built for malignancy and metastasis signatures using survminer R package. All p-values were corrected for multiple-testing hypotheses using the Benjamini-Hochberg (False Discovery Rate) method. 345 patients from TCGA-SKCM were analyzed, after removing individuals with missing data.

### RNA expression

RNA sequencing of the cell lines melan-a, 4C, 4C11− and 4C11+ was performed in triplicates in the Illumina HiSeq 1500 platform. Analysis of the data was conducted by trimming of the fragments using Trimmomatic [67], STAR alignment [68], and Rsubread [69] counting of the fragments, to assess the expression level of RNA in each of the cell lines. A complete description of the RNA-seq analysis can be found in Pessoa and colleagues [16].

## Supplementary Information

**Additional file 1: Table S1.** Complete list of differentially methylated CpGs with the respective genes for each pairwise comparison.

**Additional file 2: Table S2.** Genes with differentially methylated promoters in the malignancy and metastasis signatures.

**Additional file 3: Table S3.** Characteristics of the melanoma patients from TCGA-SKCM selected for the Cox proportional-hazards models.

**Additional file 4: Table S4.** Cox proportional-hazards models of significant hypermethylated and hypomethylated CpGs individually from malignancy and metastasis signatures.

**Additional file 5: Table S5.** Complete list of covariates included in the final Cox survival model for malignancy and metastasis signatures with their respective metrics.

**Additional file 6: Table S6.** Equivalence list between mouse ERRBS CpGs location and human Infinium 450 k CpGs probes and location referenced by their closest gene.

**Additional file 7: Figure S1.** Distribution of different percentages of methylation across all CpGs analyzed in a sample. Three biological replicates of each cell line have their methylation levels represented.

**Additional file 8: Figure S2.** Heatmaps with differentially methylated CpGs between cell lines in the melanoma progression model. Results from pairwise comparisons between non-malignant melan-a melanocytes and non-metastatic (4C11−) and metastatic (4C11+) melanoma cell lines, between 4 and 4C11− and 4C11+, and between 4C11− and 4C11+ are shown.

**Additional file 9: Figure S3.** *Pyroxd2* and *Ptgfrn* gene silencing is not reverted in melan-a melanocytes after 5azaCdR treatment. Melan-a cells were treated with 5azaCdR for 48 h, and the expressions of *Pyroxd2* and *Ptgfrn* were determined by RT-qPCR.

## Data Availability

CpG methylation calls are available at the following link: https://github.com/flaviaerius/methylation-data/tree/master/input/raw-methylation-data.
