# Supplementary figures and images for: Genome-wide promoter methylation profiling in a cellular model of melanoma progression reveals markers of malignancy and metastasis that predict melanoma survival
Source: Clin Epigenetics. 2022 May 23;14:68. doi: 10.1186/s13148-022-01291-x (PMC9128240; doi:10.1186/s13148-022-01291-x)

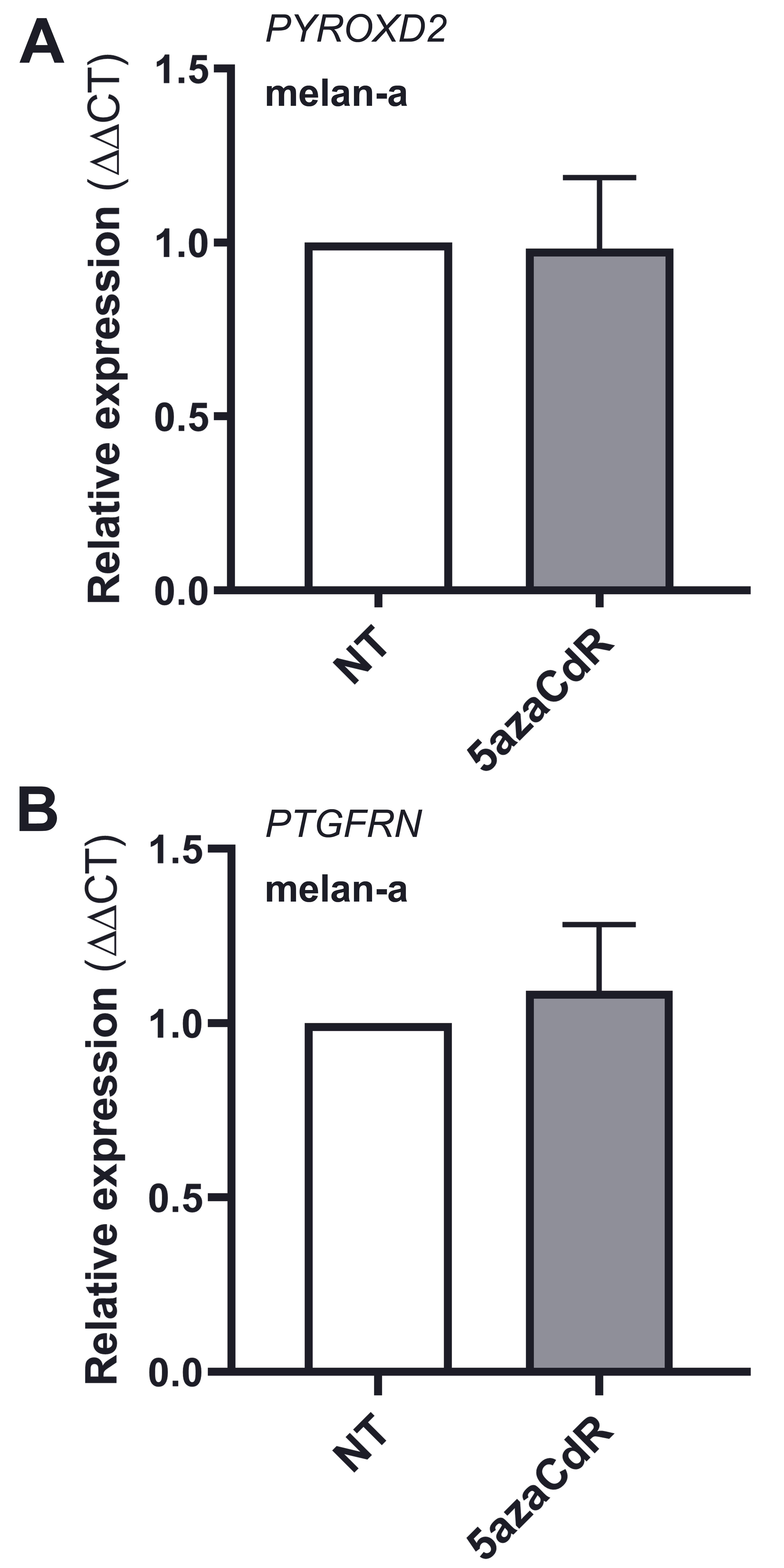

Supplement: Supplementary file 9 — Additional file 9: Figure S3. Pyroxd2 and Ptgfrn gene silencing is not reverted in melan-a melanocytes after 5azaCdR treatment. Melan-a cells were treated with 5azaCdR for 48 h, and the expressions of Pyroxd2 and Ptgfrn were determined by RT-qPCR. [file 13148_2022_1291_MOESM9_ESM.tiff]
